# The influence of cyclooxygenase inhibitors on kynurenic acid production in rat kidney: a novel path for kidney protection?

**DOI:** 10.1007/s43440-023-00460-w

**Published:** 2023-02-14

**Authors:** Izabela Zakrocka, Wojciech Załuska

**Affiliations:** Department of Nephrology, Medical University of Lublin, Jaczewskiego 8, 20-954 Lublin, Poland

**Keywords:** Kynurenic acid, Kynurenine, Tryptophan, Kidney, Prostaglandins, Cyclooxygenase, Non-steroidal anti-inflammatory drugs, Acetaminophen

## Abstract

**Background:**

Kidney diseases have become a global health problem, affecting about 15% of adults and being often under-recognized. Immunological system activation was shown to accelerate kidney damage even in inherited disorders. The kynurenine pathway is the main route of tryptophan degradation. A metabolite of kynurenine (KYN), kynurenic acid (KYNA), produced by kynurenine aminotransferases (KATs), was reported to affect fluid and electrolyte balance as a result of natriuresis induction. The accumulation of KYNA was shown in patients with impaired kidney function and its level was related to the degree of kidney damage. Cyclooxygenase (COX) inhibitors are well-known analgesics and most of them demonstrate an anti-inflammatory effect. Their main mechanism of action is prostaglandin synthesis blockade, which is also responsible for their nephrotoxic potential. Since the KYN pathway is known to remain under immunological system control, the purpose of this study was to analyze the effect of 9 COX inhibitors on KYNA production together with KATs’ activity in rat kidneys in vitro.

**Methods:**

Experiments were carried out on kidney homogenates in the presence of L-KYN and the selected compound in 6 various concentrations.

**Results:**

Among the examined COX inhibitors only acetaminophen did not change KYNA production in rat kidneys in vitro. Additionally, acetaminophen did not affect the activity of KAT I and KAT II, whereas acetylsalicylic acid and ibuprofen inhibited only KAT II. The remaining COX inhibitors decreased the activity of both KATs in rat kidneys in vitro.

**Conclusion:**

Our study provides novel mechanisms of COX inhibitors action in the kidney, with possible implications for the treatment of kidney diseases.

**Graphical abstract:**

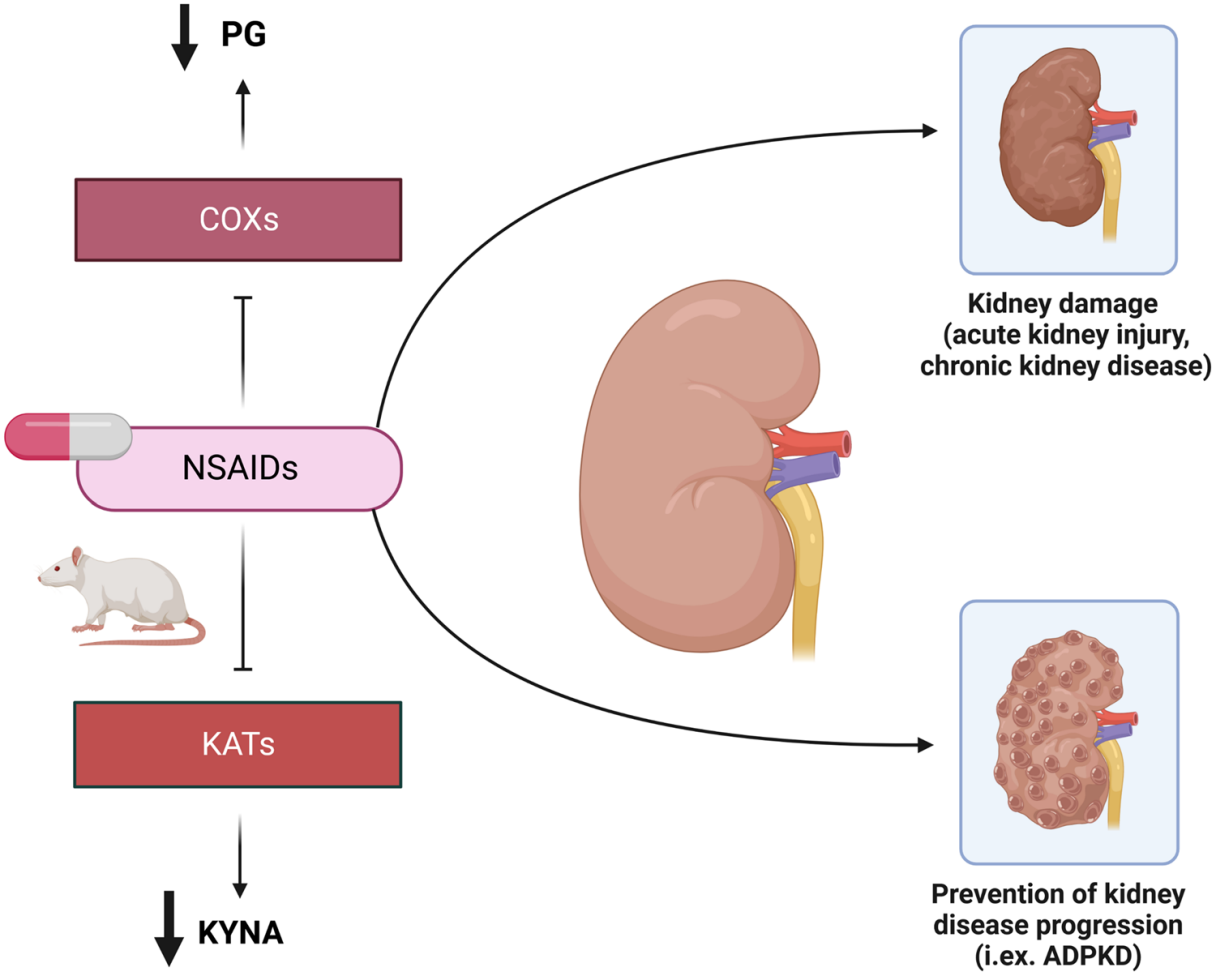

## Introduction

Kidney diseases have become a global health burden, significantly increasing cardiovascular and all-cause morbidity and mortality [1]. Chronic kidney disease (CKD), predominantly caused by diabetes mellitus, is known to affect more than 800 million individuals worldwide [2]. Due to the higher number of CKD risk factors, especially obesity, and its complications, CKD is predicted to be the fifth cause of death since 2040 [2]. The increasing prevalence of kidney disorders and their impact on global health indicate the need for exploring kidney damage mechanisms and nephroprotection methods.

Immune system dysregulation has been implicated in the pathogenesis of various kidney disorders, especially in different types of glomerulonephritis [3], acute kidney injury (AKI) [4], and CKD [5]. Interestingly, according to recently published studies, the role of inflammation in the course of metabolic or congenital diseases, like autosomal dominant polycystic kidney disease (ADPKD) [6] has been pointed out. Nonselective blockade of prostaglandin (PG) synthesis through cyclooxygenase (COX) inhibition has been reported to slow down the growth of cysts in the animal model of ADPKD [7].

Kynurenine (KYN) pathway is the main route of tryptophan degradation. Constitutive enzyme called tryptophan 2,3-dioxygenase (TDO) converts tryptophan to KYN in the liver, whereas another enzyme, indoleamine 2,3-dioxygenase (IDO), is known to be activated in response to inflammatory stimuli in various tissues [8]. In further steps, KYN is a source of biologically active compounds with pleiotropic effects [9]. Most data about the biological role of the KYN pathway are available from neurological studies, indicating the role of KYN metabolites in the pathogenesis of neurodegenerative diseases or epilepsy [10]. However, less is known about the peripheral KYN pathway activity and the mechanisms of its regulation.

A tryptophan metabolite, kynurenic acid (KYNA), is produced from KYN by kynurenine aminotransferases (KATs) [11]. KAT I and KAT II are the most thoroughly analyzed KAT isoenzymes. The antagonism towards α7-nicotinic receptors and all types of ionotropic glutamatergic receptors is known to be the predominant mechanism of KYNA’s action [12]. Additionally, KYNA was shown to affect G protein-coupled receptor (GPR)-35 and aryl hydrocarbon receptor (AhR) activity [13]. In particular, the modulation of AhR activity has recently generated a lot of interest. Although AhR activation is required for kidney development and for the maintenance of normal kidney function, increased AhR activity in the kidney was found in CKD animal models and in patients with CKD [14]. The bifunctional role of AhR, especially as a regulator of oxidative reactions, has been widely studied [15]. AhR activation was found to increase the expression of COX-2 [16], whereas in the AhR knockout mice model of diabetic nephropathy, COX-2 activity and PG production was significantly lower, together with decreased lipid peroxidation, oxidative stress level, and extracellular matrix accumulation [17]. On the other hand, some nonselective COX inhibitors, diclofenac [18] and sulindac [19] have been presented as AhR ligands that decreased renal perfusion and promoted kidney damage in healthy subjects and patients with impaired kidney function, respectively.

In previous studies, KYNA was reported to have a natriuretic [20] and chronotropic negative effect [21] in the animal model of hypertension. However, there is a growing body of evidence suggesting the relationship between KYNA level and the degree of kidney damage. Higher KYNA serum concentration, together with KYN and quinolinic acid, were related to CKD severity and the concentration of inflammatory markers [22]. Previously two classes of drugs, angiotensin-converting enzyme (ACE) inhibitors [23] and angiotensin II type 1 receptor blockers (ARBs) [24] were shown to inhibit KYNA production in rat kidneys in vitro.

Based upon previous findings that KYN pathway activity is under the influence of the immune system and that COX inhibitors are known to impair kidney function, the goal of this study was to determine the effect of the most commonly used COX inhibitors, called nonsteroidal anti-inflammatory drugs (NSAIDs): acetylsalicylic acid, diclofenac, ibuprofen, indomethacin, meloxicam, naproxen, nimesulide, piroxicam, and acetaminophen, on KYNA production and KATs activity in rat kidney in vitro.

## Materials and methods

### Animals

Presented experiments were performed on 28 male Wistar rats housed in the Experimental Medicine Center, Lublin, Poland. Animals were kept in the laboratory minimum of 7 days before planned tests were performed. Rats weighing 150–200 g were stored under standard laboratory conditions (temperature 21 °C ± 1 °C, 55 ± 5% humidity, 12 h light/dark cycle) with chow and water available ad libitum. All experiments were performed between 7 a.m. and 1 p.m. The study was carried out in accordance with the European Directive 2010/63/EU on the protection of animals used for scientific purposes. Animal tissues were obtained based on the Local Ethics Committee for Animal Experiments in Lublin approval (No. 32/2014 of 13 June 2014).

### Chemical substances

L-Kynurenine sulfate salt (K3750), acetaminophen (A7085), acetylsalicylic acid (A5376), diclofenac sodium salt (D6899), ibuprofen (I4883), indomethacin (I7378), meloxicam sodium salt hydrate (M3935), naproxen sodium (M1275), nimesulide (N1016), piroxicam (P5654), reagents for Krebs Ringer buffer preparation: sodium chloride (S7653), potassium chloride (P9333), magnesium sulfate heptahydrate (M7506), calcium chloride anhydrous (C1016), sodium phosphate monobasic dihydrate (71,505), sodium phosphate dibasic (S0876), glucose (G8270); dimethyl sulfoxide (DMSO) (D1435); reagents for KATs analysis: Trizma base (T1503), acetic acid (A6283), pyridoxal 5′-phosphate hydrate (P9255), 2-mercaptoethanol (M3148), sodium pyruvate (P2256), and D-glutamine (D9003) were obtained from Sigma-Aldrich. Substances needed to perform high-performance liquid chromatography (HPLC) were purchased from J.T. Baker Chemicals and from Sigma-Aldrich. Most tested drugs were dissolved in DMSO, whereas naproxen was administered in an aqueous solution. DMSO was given to adequate control samples and its concentration was not higher than 5% [25].

### The procedure of KYNA synthesis analysis in rat kidney in vitro

In the first step, after the animals were decapitated, rat kidneys were harvested and immediately put on ice. Afterward whole kidneys were weighed and homogenized in prepared oxygenated Krebs–Ringer buffer at pH 7.4 (1:4; w/v). 100 µL of kidney homogenate was put into test tubes, pre-filled with oxygenated Krebs–Ringer buffer (containing 800 μL in every tube). Then, the homogenate was incubated for 2 h at 37 °C together with 10 µM L-KYN (50 µL) and one of the tested COX inhibitors: acetaminophen, acetylsalicylic acid, diclofenac, ibuprofen, indomethacin, meloxicam, naproxen, nimesulide or piroxicam (50 µL). L-KYN concentration used in this experimental procedure was higher than in KATs activity analysis, due to lower basal KYNA production by kidney homogenates in vitro. Six various drug concentrations were analyzed in the study: 1 μM, 10 μM, 50 μM, 100 μM, 500 μM, and 1 mM. At least six independent kidney samples were used for each experiment in this part of the study. Control samples, instead of the drug solution, contained an equal volume of a drug solvent (50 µL of DMSO or water). The reaction was stopped by adding 1 N HCl (100 μL per sample) on ice. After that, all samples were centrifuged (15,133×*g*, 15 min), and the supernatants were collected and subjected to the HPLC analysis (Thermo Fisher Scientific HPLC system, ESA catecholamine HR-80, 3 μm, C18 reverse-phase column, mobile phase 250 mM zinc acetate, 25 mM sodium acetate, 5% acetonitrile, pH 6.2, flow rate 1.0 mL/min; fluorescence detector parameters: excitation 344 nm, emission 398 nm), and KYNA level was quantified fluorometrically. To achieve comparable results, each experiment was repeated twice. Tissues from 14 animals were used in this part of the study.

### The procedure of KATs activity analysis in rat kidney in vitro

KAT I and KAT II activity in rat kidneys in vitro was analyzed based on procedures previously presented in a study by Gramsbergen et al. [26]. In brief, kidneys were homogenized in dialysate buffer (1:9; w/v) prepared from 5 mM Tris–acetate buffer (pH 8.0), 50 μM pyridoxal 5′-phosphate and 10 mM 2-mercaptoethanol. The homogenate was centrifuged (15,133×g, 15 min) and then collected supernatant was dialyzed against 4 L of the dialysate buffer for 12 h at 8 °C with the use of cellulose membrane dialysis tubing. Afterward, the obtained enzyme sample was incubated for 2 h at 37 °C with L-KYN (2 μM) and the tested drugs at 6 different concentrations (1 μM, 10 μM, 50 μM, 100 μM, 500 μM, and 1 mM). L-KYN concentration used in this analysis was equal to plasma KYN concentration, as reported by Pawlak et al. [27], and was sufficient to obtain appropriate enzymatic activity. The optimal pH was set at 9.5 and 7.0 for KAT I or KAT II activity analysis, respectively. Glutamine (2 mM), a KAT I inhibitor, was added to samples to measure KAT II’s activity. The reaction was terminated by moving all samples into the ice-cold bath. Finally, all samples were centrifuged and analyzed by HPLC, as described in the previous section. All assays were carried out in triplicates to get comparable results. To analyze KAT’s activity, kidneys from 14 rats were used.

### Statistical analysis

Presented data are shown as mean ± standard deviation (SD). The one-way analysis of variance (one-way ANOVA) followed by Tukey’s multiple comparison test was applied to analyze differences between tested COX inhibitors. The half-maximal inhibitory concentrations values (IC50) were assessed by fitting the experimental data to a four-parameter logistic equation. Statistical analyses were done with the use of GraphPad Prism 6. Values of *p* < 0.05 were considered to be statistically significant.

## Results

### The influence of COX inhibitors on KYNA production in rat kidney in vitro

**Fig. 1:**
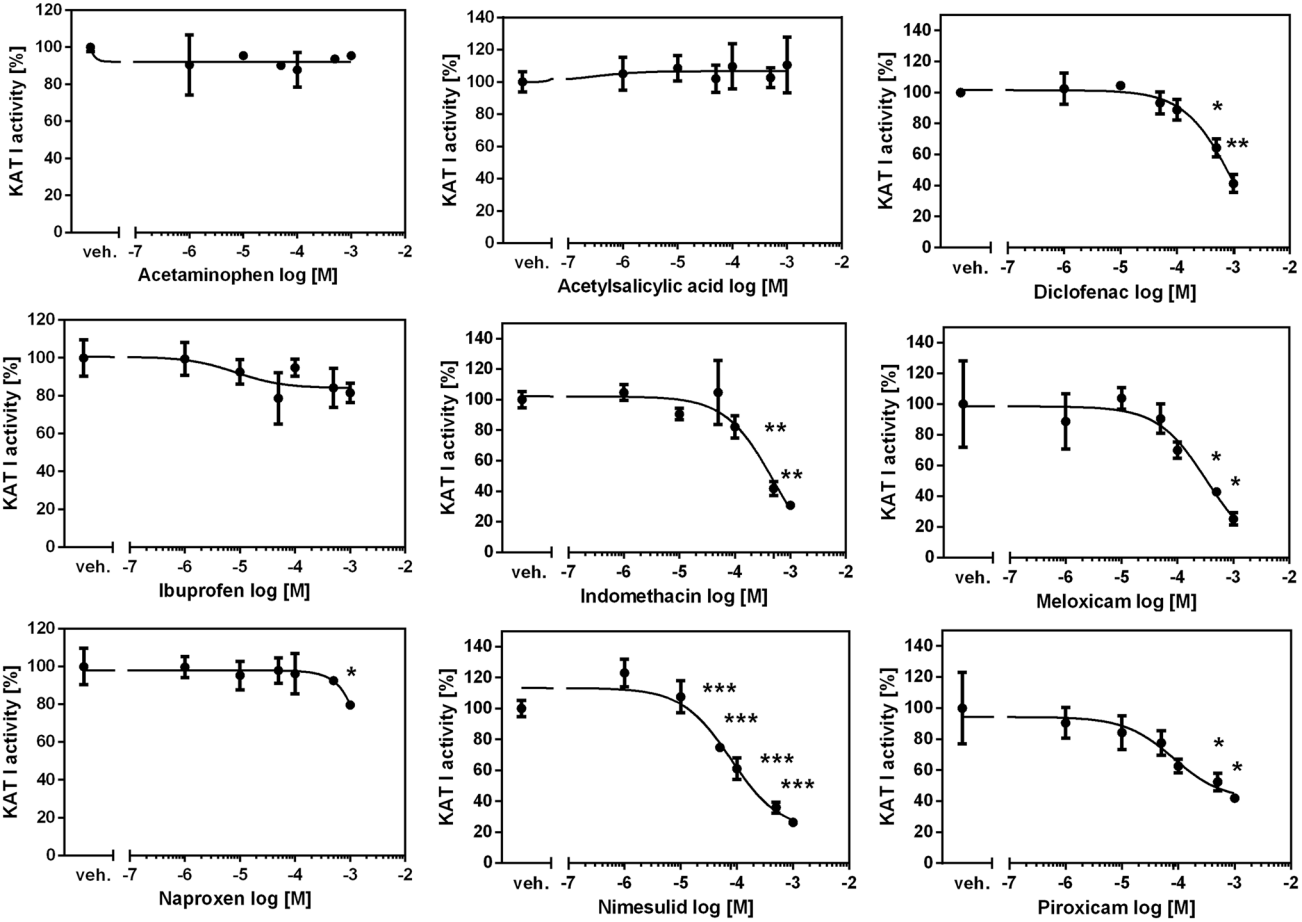
The effect of COX inhibitors on KYNA production in rat kidney in vitro. ANOVA followed by Tukey’s multiple comparison test. The data are shown as a percentage of control KYNA production, mean ± SD, *n* = 6, veh.—vehicle, *p < 0.05, **p < 0.01, ***p < 0.001

Basal production of KYNA in rat kidney homogenate in the presence of 10 μM KYN was 4.52 ± 1.73 pmol/mg tissue. Diclofenac and indomethacin were the strongest KYNA production inhibitors among the examined drugs. Diclofenac decreased KYNA synthesis by 64% with IC50 of 230 μM (*F*_5,30_ = 39.05, *p* < 0.0001) (Fig. 1). Indomethacin showed similar inhibitory activity with IC50 of 246 μM (*F*_5,30_ = 2.952, *p* = 0.0284). Naproxen displayed lower inhibitory activity with IC50 of 464 μM (*F*_5,30_ = 15.14, *p* < 0.0001). Similarly, nimesulide and piroxicam showed inhibitory activity with IC50 value of 473 μM (*F*_5,30_ = 31.87, *p* < 0.0001) and 474 μM (*F*_5,30_ = 18.12, *p* < 0.0001), respectively. Acetylsalicylic acid (F_5,30_ = 12.78, *p* < 0.0001), ibuprofen (*F*_5,30_ = 13.34, *p* < 0.0001) and meloxicam (*F*_5,30_ = 9.126, *p* < 0.0001) were the weakest KYNA synthesis inhibitors, with IC50 exceeding 1 mM. Acetaminophen did not change KYNA production in rat kidney in vitro (*F*_5,30_ = 2.081, *p* = 0.0998).

### The influence of COX inhibitors on KAT I activity in rat kidney in vitro

**Fig. 2:**
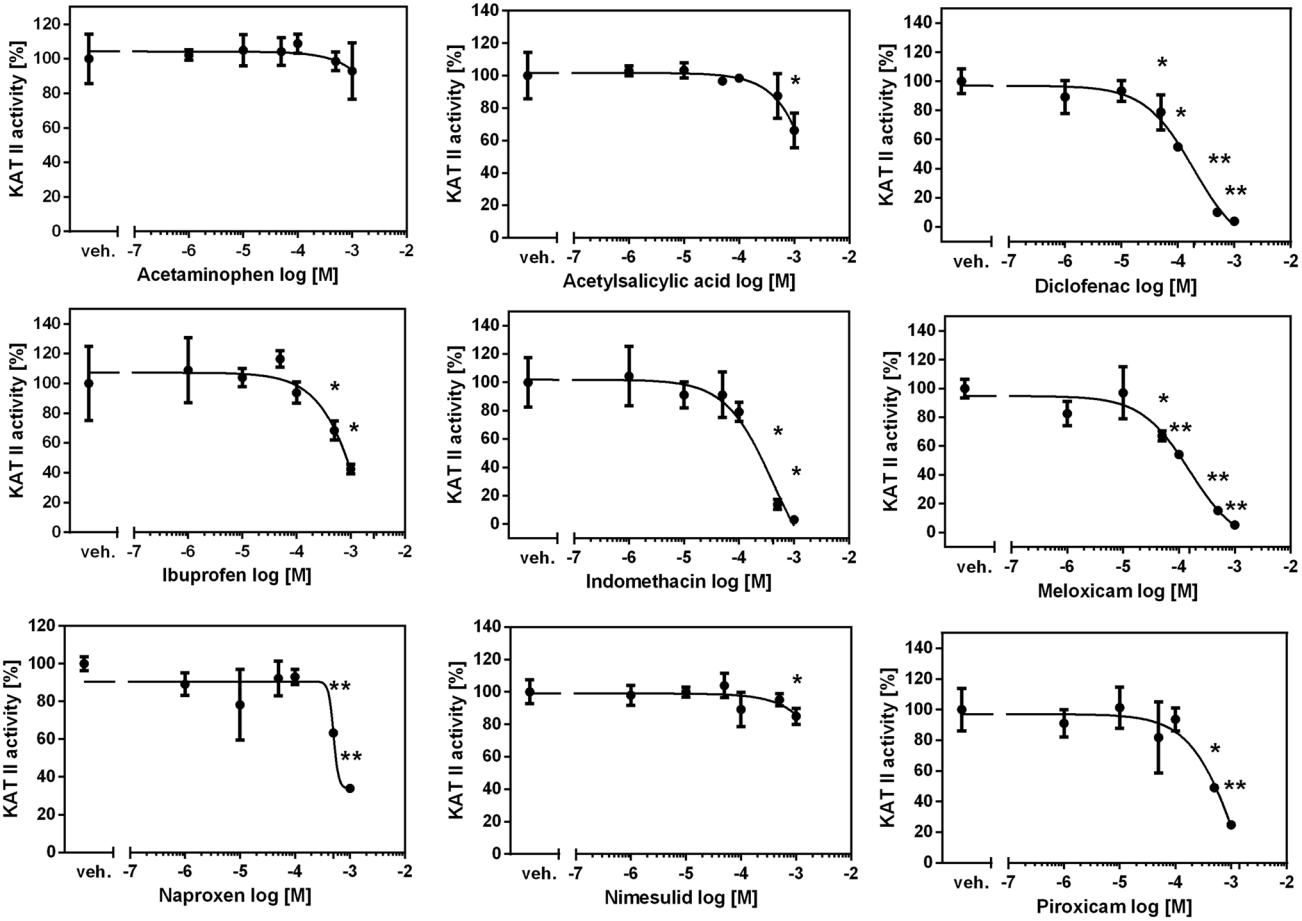
The effect of COX inhibitors on KAT I activity in rat kidney in vitro. ANOVA followed by Tukey’s multiple comparison test. The data are shown as a percentage of control KAT I activity, mean ± SD, *n* = 3, veh.—vehicle, *p < 0.05, **p < 0.01, ***p < 0.001

Standard KAT I activity in rat kidney in vitro in the presence of 2 µM KYN was 52.18 ± 18.43 pmol of KYNA/mg tissue. Nimesulide was the strongest inhibitor of KAT I in the kidney with IC50 of 128 µM (*F*_5,12_ = 58.59, *p* < 0.0001) (Fig. 2). Meloxicam and indomethacin decreased less KAT I activity in rat kidney homogenates in vitro*,* with IC50 of 310 µM (*F*_5,12_ = 33.06, *p* < 0.0001) and 503 µM (*F*_5,12_ = 32.92, *p* < 0.0001), respectively. Diclofenac (*F*_5,12_ = 41.55, *p* < 0.0001), piroxicam (*F*_5,12_ = 19.67, *p* < 0.0001) and naproxen (*F*_5,12_ = 3.746, *p* = 0.0283) displayed higher IC50 values above 1 mM. Acetaminophen (*F*_5,12_ = 0.5384, *p* = 0.7439), acetylsalicylic acid (*F*_5,12_ = 0.3192, *p* = 0.8920), and ibuprofen (*F*_5,12_ = 2.765, *p* = 0.0691) did not affect the activity of KAT I in rat kidney in vitro.

### Influence of COX inhibitors on KAT II activity in rat kidney in vitro

**Fig. 3:**
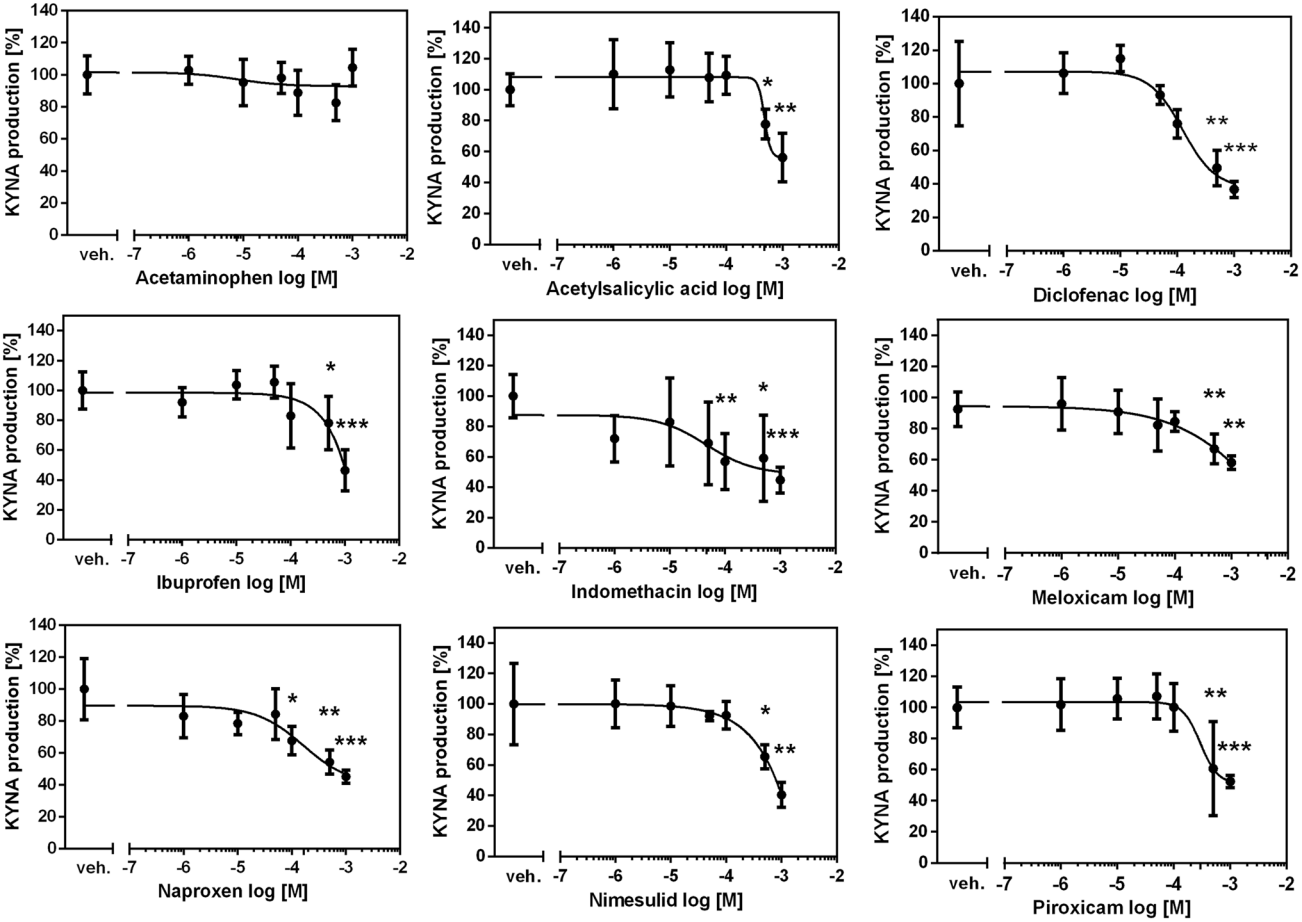
The effect of COX inhibitors on KAT II activity in rat kidney in vitro. ANOVA followed by Tukey’s multiple comparison test. The data are shown as a percentage of control KAT II activity, mean ± SD, *n* = 3, veh.—vehicle, *p < 0.05, **p < 0.01

The mean KYNA production in rat kidneys in vitro by KAT II under 2 µM L-KYN was 91.05 ± 30.47 pmol/mg tissue. With IC50 of 155 μM, meloxicam was the most potent KAT II inhibitor in rat kidneys in vitro (*F*_5,12_ = 58.62, *p* < 0.0001) (Fig. 3). Similar inhibitory activity was displayed by diclofenac with IC50 of 191 µM (*F*_5,12_ = 85.60, *p* < 0.0001). Indomethacin and naproxen blocked KAT II with IC50 equal to 420 µM (*F*_5,12 =_ 40.98, *p* < 0.0001) and 504 μM (*F*_5,12_ = 19.58, *p* < 0.0001), respectively. Piroxicam (*F*_5,12_ = 18.85, *p* < 0.0001), ibuprofen (*F*_5,12_ = 22.17, *p* < 0.0001), nimesulide (*F*_5,12_ = 3.480, *p* = 0.0356) and acetylsalicylic acid (*F*_5,12_ = 10.69, *p* = 0.0004) were the weakest KAT II inhibitors, with IC50 values exceeding 1 mM. Acetaminophen was not shown to inhibit KAT II activity in rat kidneys in vitro (*F*_5,12_ = 1.419, *p* = 0.2858).

## Discussion

The presented study demonstrates for the first time the influence of various COX inhibitors on KYNA production in rat kidneys in vitro. We found that most of the drugs under examination, except acetaminophen, decrease KYNA production in rat kidneys in vitro. Diclofenac was the strongest KYNA synthesis inhibitor in the kidney, whereas other COX inhibitors displayed lower inhibitory capacity (indomethacin > naproxen > nimesulide > piroxicam > acetylsalicylic acid > ibuprofen > meloxicam). In addition to that, significant KAT I inhibition by tested COX inhibitors, apart from acetaminophen, acetylsalicylic acid, and ibuprofen, was shown (nimesulide > meloxicam > indomethacin > diclofenac > piroxicam > naproxen). Similarly, all analyzed drugs, except acetaminophen, reduced KAT II activity in rat kidneys in vitro, with meloxicam and diclofenac as the strongest KAT II inhibitors, and other COX inhibitors presenting lower KAT II inhibitory activity (indomethacin > naproxen > piroxicam > ibuprofen > nimesulide > acetylsalicylic acid).

PGs are major metabolites of arachidonic acid produced by COX, responsible for triggering inflammatory responses [28]. The main PG, PGE2, was shown to be involved in renal hemodynamics, renin release, and tubular sodium with water absorption [29, 30]. Increased PG production was observed in various kidney diseases, especially in diabetic kidney disease [31], glomerulonephritis [32] or ADPKD [33]. COX-1 isoenzyme is claimed to be constitutively expressed, whereas COX-2 expression is induced under inflammatory conditions [34], and also in chronic sodium deficiency or excessive ultrafiltration [35]. Interestingly, it was pointed out that COX-2 is also constitutively expressed in the kidney, suggesting that its inhibition can significantly impair kidney function [36]. NSAIDs were shown to inhibit both COX isoenzymes activity [37].

Chronic inflammation was reported to be tightly connected with kidney diseases, especially CKD. Schefold et al. indicated a correlation between IDO activation, disease severity and inflammatory parameters [22]. Similar observations were made by Pawlak et al., who observed higher tryptophan degradation and KYN production in CKD patients, together with increased oxidative stress parameters [38]. Among KYN metabolites KYNA was recently shown to be positively correlated with CKD severity in adults with ADPKD [39]. Furthermore, Dąbrowski et al. reported that only plasma KYNA concentration was correlated with the level of procalcitonin and lactate level in patients with septic shock and AKI, predicting their survival [40]. Based on these findings, it should be concluded that modulating KYNA synthesis can be an interesting tool for the prevention and treatment of kidney diseases.

Based on available studies, which have given a novel insight into the inflammatory pathogenesis of kidney diseases, the inhibition of KYNA synthesis by COX inhibitors can improve kidney function, especially in ADPKD. PGs, as well as other inflammation markers, were shown to stimulate cell proliferation, the growth of cysts, and fluid secretion in primary cultured ADPKD cells [41]. Nonselective COX inhibition by sulindac [7] and selective COX-2 blockade by celecoxib [42] were reported to decrease cysts volume in animal models of ADPKD. An interesting view on the association between KYN pathway activity and ADPKD severity was recently shown by Klawitter et al., who found KYNA to be positively correlated with disease severity, expressed as height-adjusted kidney volume and estimated glomerular filtration rate [39]. According to this study, inhibition of KYNA synthesis should be considered a novel method of slowing ADPKD progression. Angiotensin-converting enzyme (ACE) inhibitors and angiotensin II type 1 receptor blockers (ARBs) are already known to have an anti-inflammatory effect, to decrease kidney damage in ADPKD [43] and lower KYNA synthesis in the kidney [23, 24]. Based on our study, COX inhibitors, through KYNA production inhibition, can be considered as potential drugs for ADPKD treatment and other diseases of immune-mediated origin. Adding to that, there are available reports suggesting kidney protection by COX inhibitors in an animal model of diabetes [44] or sepsis-induced AKI [45].

In the presented study, among tested COX inhibitors naproxen, diclofenac and indomethacin have been shown as the strongest inhibitors of KYNA synthesis in the kidney. Moreover, these drugs caused significant inhibition of KAT II, the crucial enzyme involved in KYNA production. Since the aforementioned drugs are known to cause kidney damage, especially through impaired renal hemodynamics [46], the results of our study indicate a novel possible mechanism of nephrotoxicity caused by selected COX inhibitors. In previous studies, KYNA was found to attenuate kidney injury in an animal model of heat stroke [47] or ischemia reperfusion-induced AKI [48]. In this manner, the inhibition of KYNA production by COX inhibitors should be considered potentially toxic.

Adding to that, other risks, including increased mortality, should be considered in relation to the administration of COX inhibitors. In a systematic review published by Asghar and Jamali, an analysis was performed to stratify cardiovascular and renal risks of meloxicam use compared with other NSAIDs [49]. Interestingly, meloxicam demonstrated a lower risk of vascular complications and no risk of renal episodes, adversely to other COX inhibitors, indomethacin, diclofenac, naproxen, and ibuprofen, which were shown to increase the risk of renal side effects and all-cause mortality [49]. Similarly, in a Danish nationwide cohort study by Schmidt et al. diclofenac posed the highest cardiovascular risk, compared with other NSAIDs and acetaminophen [50]. On the other hand, weaker KYNA synthesis inhibitors, acetylsalicylic acid, ibuprofen and meloxicam should be considered as less nephrotoxic drugs. Indeed, long-term acetylsalicylic acid administration was reported to not affect kidney function in a randomized controlled trial in patients with diabetes mellitus [51].

COX-3 isoenzyme, a splice variant of COX-1, was previously considered as a place of action of acetaminophen, explaining different properties of this drug [52]. However, since COX-3 was not found in humans, other mechanisms, including the inhibition of peroxidase (POX) site by acetaminophen was suggested [52]. Similarly in our study, acetaminophen showed distinct effect on KYNA synthesis in the kidney than the rest of COX inhibitors. Since acetaminophen did not change the activity of KATs and KYNA production, it can be suggested that this drug has weaker effect on kidney function impairment compared with the other COX inhibitors being analyzed, as suggested by [53].

Partly similar results indicating the effect of COX inhibitors on KYNA production were shown previously, although not in kidney tissue. Decreased KYNA content in the rat brain after meloxicam administration was presented in a study by Schwieler et al., whereas diclofenac and indomethacin elevated KYNA levels [54]. Similarly, diclofenac increased KYNA concentration in the rat brain following tail ischemia, suggesting KYNA increase being responsible for the analgesic effect of diclofenac [55]. It should be emphasized that the different effects of diclofenac on KYNA production, compared to our study, can be related to various experimental protocols and the type of organs studied. Recently, Savitz et al. presented a randomized, placebo-controlled, crossover study on 20 healthy volunteers, indicating that the acute administration of ibuprofen increases serum KYNA concentration, although 5 h after drug administration [56].

Our study has its limitation. We have shown the effect of 9 COX inhibitors in 6 different concentrations, reaching up to 1 mM to check how efficiently these drugs can influence KYNA production in a dose-dependent manner. After oral administration, selected drugs showed inhibitory effect at concentrations exceeding those reported in the rat serum [57]. However, most available studies on COX inhibitors pharmacokinetics were performed on isolated rat kidneys, using various concentrations of drugs ranging from 12 to 120 µM in the ibuprofen study [58] up to 10 mM in the acetaminophen study [59].

## Conclusions

The presented study indicates a novel mechanism of action of COX inhibitors. The reduction of KYNA production and KATs activity in the kidney by COX inhibitors provides another pathway for the prevention and treatment of inflammatory-mediated kidney diseases. The potentially unfavorable effect of COX inhibitors, related to the inhibition of KYNA synthesis and KATs activity in the kidney should also be considered.

## Data Availability

The datasets generated during and/or analyzed during the current study are available from the corresponding author upon reasonable request.

## Abbreviations

ACE: Angiotensin-converting enzyme
ADPKD: Autosomal dominant polycystic kidney disease
AhR: Aryl hydrocarbon receptor
AKI: Acute kidney injury
ARB: Angiotensin II type 1 receptor blocker
CKD: Chronic kidney disease
COX: Cyclooxygenase
DMSO: Dimethyl sulfoxide
GPR: G protein-coupled receptor
HPLC: High-performance liquid chromatography
IC50: Half-maximal inhibitory concentration
IDO: Indoleamine 2,3-dioxygenase
KAT: Kynurenine aminotransferase
KYN: Kynurenine
KYNA: Kynurenic acid
NSAID: Nonsteroidal anti-inflammatory drug
PG: Prostaglandin
TDO: Tryptophan 2,3-dioxygenase
